# Mechanistic Insights From Single-Molecule Studies of Repair of Double Strand Breaks

**DOI:** 10.3389/fcell.2021.745311

**Published:** 2021-11-15

**Authors:** Muwen Kong, Eric C. Greene

**Affiliations:** Department of Biochemistry and Molecular Biophysics, Columbia University Irving Medical Center, New York, NY, United States

**Keywords:** non homologous end joining (NHEJ), homologous recombination (HR), single-molecule, DNA repair, optical tweezers (OT), magnetic tweezers, DNA curtain, FRET—fluorescence resonance energy transfer

## Abstract

DNA double strand breaks (DSBs) are among some of the most deleterious forms of DNA damage. Left unrepaired, they are detrimental to genome stability, leading to high risk of cancer. Two major mechanisms are responsible for the repair of DSBs, homologous recombination (HR) and nonhomologous end joining (NHEJ). The complex nature of both pathways, involving a myriad of protein factors functioning in a highly coordinated manner at distinct stages of repair, lend themselves to detailed mechanistic studies using the latest single-molecule techniques. In avoiding ensemble averaging effects inherent to traditional biochemical or genetic methods, single-molecule studies have painted an increasingly detailed picture for every step of the DSB repair processes.

## Introduction

Maintenance of genome stability is paramount to the survival of all living organisms. Both extrinsic environmental factors, as well as intrinsic, routine cellular processes such as transcription and replication can lead to DNA damage and contribute to genome instability. Understanding DNA damage and genome maintenance is a crucial aspect of cancer research, as they are involved in carcinogenesis and cancer therapies (Hoeijmakers, 2009).

Though accounting for only 0.01% of the ∼10^5^ spontaneous DNA lesions that a cell experiences per day, double strand breaks (DSBs) pose a unique challenge to repair in that the physical continuity of the DNA molecule is disrupted (Lindahl, 1993; Vilenchik and Knudson, 2003). DSBs can arise from a variety of exogenous factors such as ionizing radiation and chemotherapeutic drugs, as well as endogenous sources such as replication stress, V(D)J recombination, and meiosis. In addition, DSBs can also be generated when single strand breaks (SSBs), which are much more common, are encountered by DNA replication forks (Ohnishi et al., 2009). Mis-repair of DSBs can lead to deleterious consequences, causing large-scale chromosome rearrangements or local genetic mutations (Aparicio et al., 2014). Therefore, the repair process of DSBs is tightly controlled, employing complementary pathways consisted of intricately linked and carefully orchestrated steps.

Two major, well conserved, pathways in DSB repair are homologous recombination (HR) and canonical nonhomologous end joining (NHEJ). Together with pathways of alternative end joining (alt-NHEJ) and single-strand annealing (SSA), these four mechanisms are tasked to minimize undesired loss of genetic information in the process of restoring the physical continuity of DNA. Canonical and alternative end joining repair pathways directly join and ligate the two broken ends of a DSB after minimal end processing (Lieber, 2010). As these pathways require little to minimal sequence context, NHEJ and alt-NHEJ have typically been viewed as error-prone in repair. In contrast, homologous recombination is based on the search and pairing of the broken DNA end(s) to existing homologous sequence elsewhere in the genome, thus maximizing repair fidelity (San Filippo et al., 2008). HR is cell cycle dependent and takes place in S and G2 phases, when homologous sequences in sister chromatids are available as repair templates. Whereas the end-joining pathways remain functional throughout the cell cycle (Symington and Gautier, 2011).

Over the past two and half decades, single-molecule microscopy and spectroscopy have made significant contributions to characterizations of systems previously considered intractable, thanks in no small part to technological innovations in fields from physics to nanotechnology and protein engineering. In this review, we begin with a brief description of single-molecule techniques frequently used in the studies of protein-DNA interactions. The sections that follow will be dedicated to homologous recombination and nonhomologous end joining, where we first provide an overview of each of these pathways in mammalian cells. We highlight and discuss in detail the findings from single-molecule studies that contributed to mechanistic understanding of steps involved in each repair mechanism. While the focus is on eukaryotic DSB repair, insights from pioneering studies of bacterial repair proteins will also be presented when appropriate.

## Overview of Single-Molecule Techniques

A crucial hurdle that all *in vitro* single-molecule imaging studies of protein-DNA interactions must overcome is that as flexible polymers, DNA molecules, especially those significantly longer than their persistence length, collapse into random coils in the absence of external forces on the ends. Under most circumstances, unambiguous characterization of protein-DNA transactions is only possible when imaging is unencumbered by the presence of multiple DNA segments in close vicinity. To that end, several experimental approaches have been developed to maintain extended conformation of DNA molecules by exerting forces on their ends. In the sections below, we briefly describe these implementations.

Broadly speaking, there are two strategies for extending single DNA molecules to a desired end-to-end distance: mechanical force extension, typically through the use of optical or magnetic tweezers, and hydrodynamic force extension.

**FIGURE 1:**
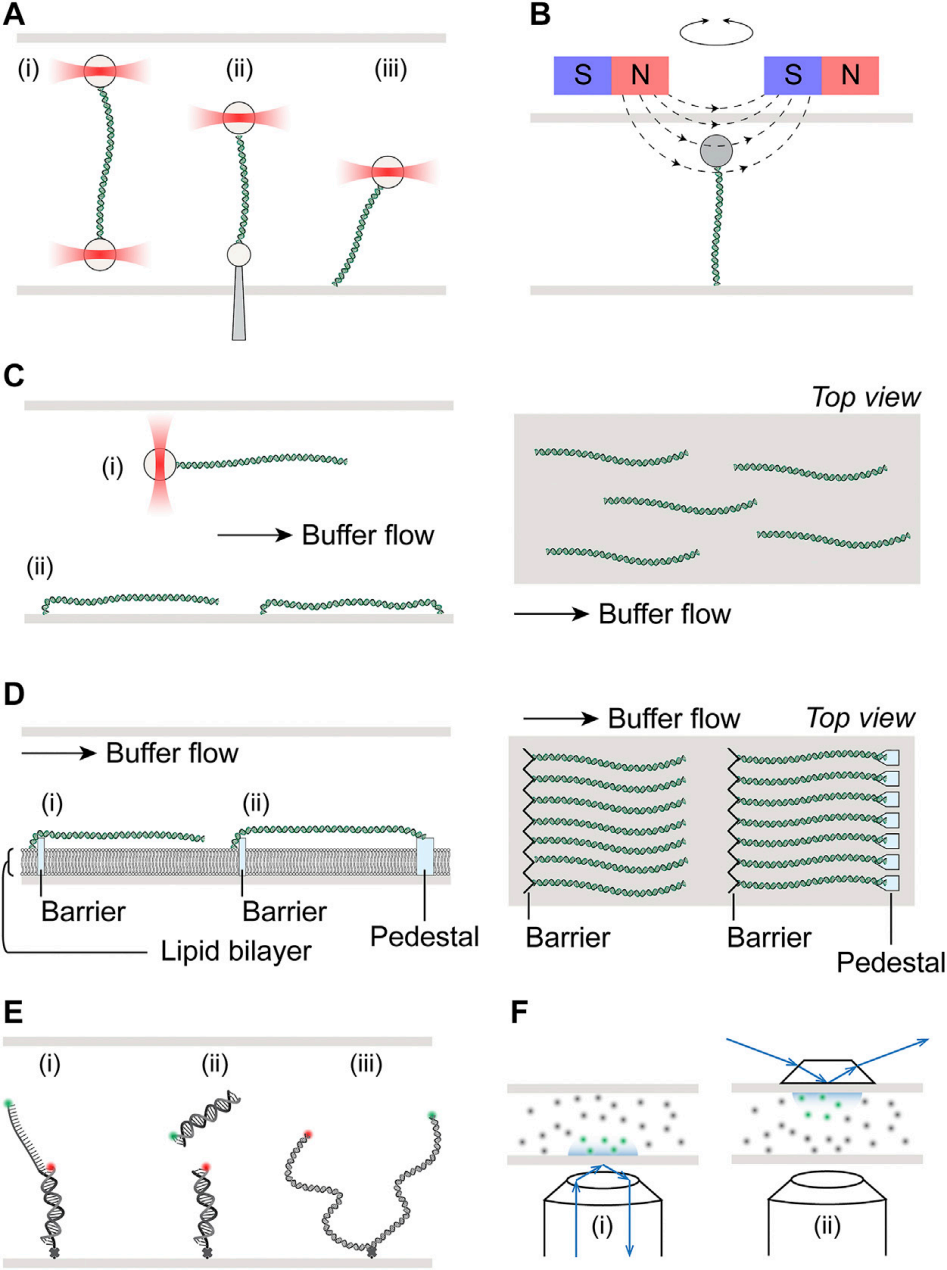
Schematics of single-molecule setups. **(A)** Schematic illustration of optical tweezers, where a single DNA molecule is tethered at one end to an optically trapped bead, and the other end to **(i)** another bead held in a second optical trap, **(ii)** a micro-pipette fixed to the flow cell, or **(iii)** the surface of the flow cell. **(B)** Schematic illustration of magnetic tweezers, where a single DNA molecule is held between a magnetic bead and the flow cell surface. Torsional stress can be applied by rotation of the external magnets. **(C)** **Left:** Hydrodynamic flow extension of DNA molecules attached to either **(i)** an optically trapped bead or **(ii)** the flow cell surface. DNA molecules may be tethered to the surface at both ends if desired. **Right:** Top view of flow extended and surface tethered DNA in a flow cell, illustrating the random spatial distribution of these molecules. **(D)** **Left:** Schematic of DNA curtains where molecules tethered at one end to the lipid bilayer are aligned at the diffusion barrier. Single-tethered DNA may be extended by buffer flow **(i)**. Alternatively, DNA maybe double-tethered at the pedestal **(ii)**. **Right:** Top view of single- or double-tethered DNA curtains, where molecules are aligned in uniformity. **(E)** Schematic illustration of smFRET, where the energy transfer may be either intramolecular **(i)** and **(iii)** or intermolecular **(ii)**. DNA molecules are immobilized on the flow cell surface via biotin-streptavidin linkage with biotin placed at the ends of short substrates **(i)** and **(ii)**, or internally for ∼kbp length substrates **(iii)**. **(F)** Schematic illustrate of total internal reflection fluorescence microscopy (TIRFM), achieved either through objective **(i)** or through prism **(ii)**. Green and gray dots represent excited inside and dark fluorophores outside the evanescence field, respectively.

Optical Tweezers (OT) is an implementation of optical manipulation that controls and measures motion of trapped microscopic dielectric particle(s) using optical/electromagnetic forces (Ashkin, 1970; Ashkin et al., 1986). Beyond its applications that led to two separate awards of the Nobel Prize in Physics (Steve Chu in 1997 and Arthur Ashkin in 2018), optical trapping has been widely adopted today as a tool to study biophysical and biochemical properties of biological macromolecules and processes (Moffitt et al., 2008; Bustamante et al., 2020). The basic principles of optical trapping involve creating a tightly focused laser beam where the spatial gradient of its intensity exerts a restoring force on an object within the beam, balancing out the scattering force that pushes the object along the direction of light propagation (Ashkin et al., 1986). While the object is near (∼150 nm) the center of the beam, the restoring force is linearly related to the displacement of the object from the center, acting as a Hookean spring (Neuman and Block, 2004). Single DNA molecules are typically extended using optical tweezers by fixing one end of the DNA to an optically trapped bead, while the other end is attached to either a physically fixed part of the flow cell assembly such as the surface or a micropipette, or another optically trapped bead (i.e., DNA dumbbells) (Figure 1A).

Similar to optical tweezers, magnetic tweezers utilize paramagnetic microspheres that are held in magnetic fields generated by external magnets (De Vlaminck and Dekker, 2012). Typically, the two ends of a DNA molecule are attached to the magnetic bead and the flow cell surface, with the molecule being extended by vertical positioning of the magnetic field relative to the flow cell (Figure 1B). While implementation of torque measurements has been developed for optical tweezers by using nanofabricated quartz cylinders held in angular optical traps (La Porta and Wang, 2004), the ability and ease to apply torque to a tethered molecule in magnetic tweezers by simply rotating the external magnetic field remain unparalleled. In addition to indirectly measuring torque through monitoring the DNA end-to-end distance, direct torque and twist measurements are also possible on magnetic tweezers with circularly symmetric or near-zero torque fields.

In comparison to mechanical force extension, hydrodynamic flow represents a more straightforward, albeit less precise, method to unravel DNA molecules. Flow stretching readily complements optical tweezers where only one end of the DNA is attached to a bead held in an optical trap, as such combination was initially used to study DNA conformational dynamics and polymer physics models (Perkins et al., 1995). When combined with surface-tethered DNA, flow stretching allows parallelization of measurements on multiple DNA molecules (Figure 1C). Briefly, one end of the DNA molecules is first immobilized on the surface of the flow cell, where they are randomly distributed spatially. These DNA molecules are then extended in the presence of applied buffer flow. Depending on the application, the down-flow ends may be left free, thus requiring continuous flow for the duration of these single-tethered experiments for real time observations. Alternatively, the second ends may also be anchored to the surface, forming double-tethered DNA and enabling steady-state observations in the absence of any buffer flow (Figure 1C). One common area of concern in these experiments is the potential of interference from the flow cell surface in protein-DNA interactions that are being studied.

A variation of surface-tethered single-molecule imaging technique named DNA curtains has been developed to minimize potential of surface interference and maximize parallelization (Graneli et al., 2006; Visnapuu et al., 2008). The platform uses nanofabricated chromium structures on flow cell surfaces to precisely align hundreds of DNA molecules at pre-determined positions (Figure 1D). Such alignment is achieved by first forming a biotinylated lipid bilayer, to which one end of the DNA molecules are tethered, on the surface of the microfluidic device. The fluidity of lipid molecule allows these single-tethered DNA molecules to diffuse freely without flow. In the presence of buffer flow, DNA molecules are pushed against the chromium diffusion barriers and uniformly extended in parallel. Furthermore, the lipid bilayer also serves as a close mimic to biological membranes in cellular environments, minimizing non-specific surface adsorption of protein or DNA. Directional double-tethering is achieved by using orthogonal attachment chemistry at both the lipid bilayer and the chromium anchors, the latter deposited a specified distance away from the alignment barriers (Figure 1D). Further development of the initial dsDNA curtain technique allowed tethering of ssDNA and greatly expanded repertoire of biological processes that could be investigated with this technology (Ma et al., 2017b; De Tullio et al., 2018).

Visualization of protein-DNA interactions on extended DNA molecules is commonly based on fluorescence microscopy. dsDNA can be visualized by staining with fluorescent intercalating dyes such as YOYO-1 or SYTOX Orange. Multiple options exist for protein labeling, including fluorescent fusion proteins, fluorescent nanocrystals (quantum dots), and a myriad of increasingly bright and photostable small-molecule fluorescent probes such as Alexa Fluor, ATTO, Janelia Fluor dyes (Ha and Tinnefeld, 2012). Fluorescent excitation may be readily accomplished *via* epi-fluorescence, total internal reflection, or confocal illumination. Each illumination scheme has its own advantages and disadvantages. For example, while total internal reflection reduces background noise significantly compared to epi-fluorescence, it is also restricted to imaging within ∼200 nm of the surface, due to the depth reachable by evanescence waves produced by total internal reflection at that surface (Selvin and Ha, 2008) (Figure 1E).

In contrast to the direct imaging approaches described above, single-molecule Forster Resonance Energy Transfer (smFRET) experiments shed light on interactions that occur at much smaller distance scale (Roy et al., 2008). smFRET monitors the distance, usually between 1 and 10 nm, between single pairs of donor and acceptor fluorophores, by measuring their intensities and the extent of non-radiative energy transfer (Ha et al., 1996). Unencumbered by diffraction limited resolution (∼250 nm) in typical fluorescence based single-molecule imaging experiments, smFRET has been widely employed in biophysical studies on topics ranging from replication, transcription and repair to RNA and protein conformational dynamics (Feng et al., 2021). *In vitro* smFRET experiments usually requires immobilization of fluorescently labeled macromolecules, either DNA or protein, on the passivated flow cell surface, where excitation of fluorophores is achieved through total internal reflection (Figure 1F). Although the length of surface-immobilized DNA used in smFRET experiments is typically short (∼100 bp), longer DNA substrates on the order of kbp have also been successful in experiments under conditions such that DNA could become chromatinzed (Graham et al., 2017).

## Overview of Homologous Recombination

**FIGURE 2:**
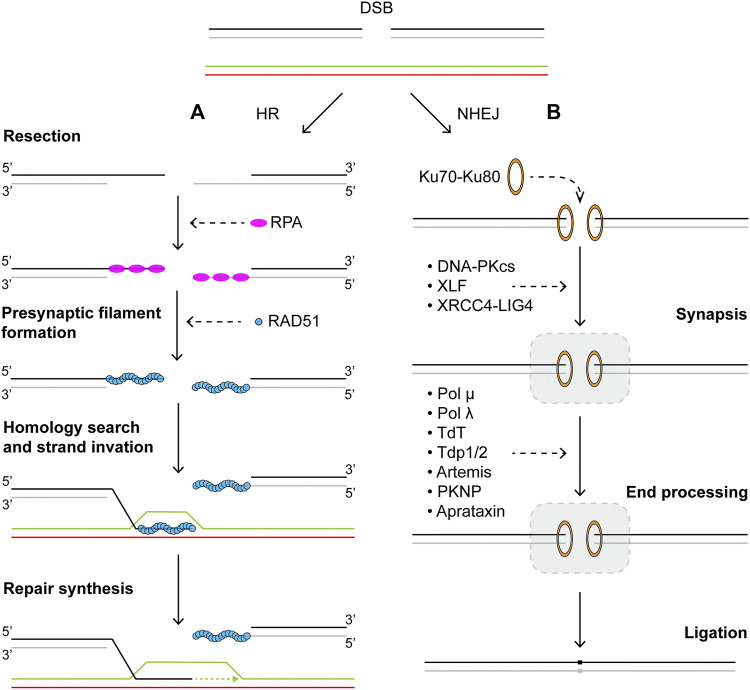
Repair of DNA double strand breaks *via* **(A)** homologous recombination or **(B)** nonhomologous end-joining pathways. Schematics of homologous recombination and nonhomologous end-joining pathways. See main text for details.

Usually considered the error-free repair pathway for DSBs, HR can be divided into four distinct stages: end resection, formation of presynaptic filament, homology search, and repair synthesis (Figure 2A). In mammalian cells, resection is initiated first by the MRN complex consisting of Mre11, Rad50, and Nbs1, in complex with CtIP (Sartori et al., 2007; Shibata et al., 2014; Anand et al., 2016). This short-range resection begins with Mre11 nicking the strand with a 5′ terminal at the break. The nick is then extended towards the break in the 3′–5′ manner by the exonuclease activity of Mre11. The single-stranded DNA gap created by short-range resection acts as a platform for long-range resection machineries to land. Proteins involved in long-range resection include EXO1, DNA2, BLM, and WRN (Eid et al., 2010; Nimonkar et al., 2011; Sturzenegger et al., 2014). EXO1 is a versatile and active 5′–3′ exonuclease. BLM and WRN are RecQ family helicases that can processively translocate on ssDNA in a 3′–5′ direction. Strand separation by BLM and WRN generates 5′ DNA flaps which are substrates for DNA2 activity. Together, their actions generate long 3′ ssDNA tails that are rapidly bound by the heterotrimeric ssDNA binding protein complex RPA to protect the integrity of DNA. Given that resection commits repair to homologous recombination, the process is subject to many forms of regulation. Phosphorylation of CtIP by CDK and ATM is essential for resection, through stimulating endonuclease activity of Mre11 as well as mediating interactions with BRCA1-BARD1 (Peterson et al., 2013; Wang et al., 2013). Furthermore, BRCA1 also plays an important role in removal of 53BP1, which is recruited to DSB sites and blocks 5′ end resection in G1 phase (Bunting et al., 2010; Mirman and de Lange, 2020). The assembly of presynaptic filament begins with binding of recombinase RAD51, homolog of bacterial RecA, to ssDNA, replacing RPA (Sung et al., 2003; Bonilla et al., 2020). Formation of the RAD51-ssDNA nucleofilament must overcome the inhibitive effects of RPA and is facilitated by recombination mediator proteins such as yeast Rad52 and human BRCA2 (Sung, 1997a; New et al., 1998; Jensen et al., 2010). BRCA2 interacts with RAD51 and together they are targeted to RPA-bound ssDNA by DSS1, a stable interaction partner of BRCA2 that also helps displacement of RPA from ssDNA. Stability of the Rad51-ssDNA filament is regulated by a number of pro- and anti-recombination proteins. Paralogues of human RAD51 form two distinct complexes, RAD51B-RAD51C-RAD51D-XRCC2 (BCDX2) and RAD51C-XRCC3 (CX3) (Masson et al., 2001a; Masson et al., 2001b). Together with the yeast complex Rad55-Rad57, as well as the Shu complexes, these paralog complexes are known to promote RAD51 filament formation and stability (Sung, 1997b; Bernstein et al., 2011; Liu T. et al., 2011; Bonilla et al., 2020). The RAD51-ssDNA nucleofilament must then undergo a homology search in an effort to locate and pair with homologous sequence elsewhere in the genome to be used as template for potentially error-free repair (Renkawitz et al., 2014; Haber, 2018). The search process is mediated by many proteins, including RAD54 (Petukhova et al., 1998; Petukhova et al., 2000; Zhang et al., 2007; Renkawitz et al., 2013). During the search, the presynaptic filament interrogates the dsDNA template and samples base pairing for homology. After recognition of homologous sequence is established, a stable heteroduplex called the displacement loop (D-loop) is formed, where the invading 3′ ssDNA tail is base paired with the complementary strand in the template DNA, displacing the homologous strand. D-loops formation, similar to that of the presynaptic filament, offers another opportunity for regulation. BRCA1-BARD1, RAD51AP1-UAF1, and PALB2 have all been shown to stimulate D-loop formation (Dray et al., 2010; Liang et al., 2016; Zhao et al., 2017). In order to initiate nascent DNA synthesis using the now paired strand as template, RAD51 is removed from the heteroduplex to expose the 3′ of the invading ssDNA, where DNA replication machineries including PCNA, RFC, and polymerase δ are assembled and can commence repair synthesis (Li et al., 2009; Sebesta et al., 2011; McVey et al., 2016). Finally, for DSBs with two free DNA ends, the repair may be completed through synthesis dependent strand annealing (SDSA), where the invading strand now extended through DNA synthesis dissociates from the D-loop structure and reanneals with the other broken end (San Filippo et al., 2008; Mehta and Haber, 2014). No crossover events occur as a result of SDSA. Alternatively, the second broken end may be captured by and annealed to the displaced strand of the D-loop, leading to the formation of a double Holliday junction (dHJ). Dissolution of dHJs by BLM and Topo IIIα will result in non-crossover events, while resolution can lead to either crossover or non-crossover events, depending on the resolvases involved (Xue et al., 2013; Bizard and Hickson, 2014; Chen et al., 2014; Matos and West, 2014).

### SM Studies of DNA End Resection

**FIGURE 3:**
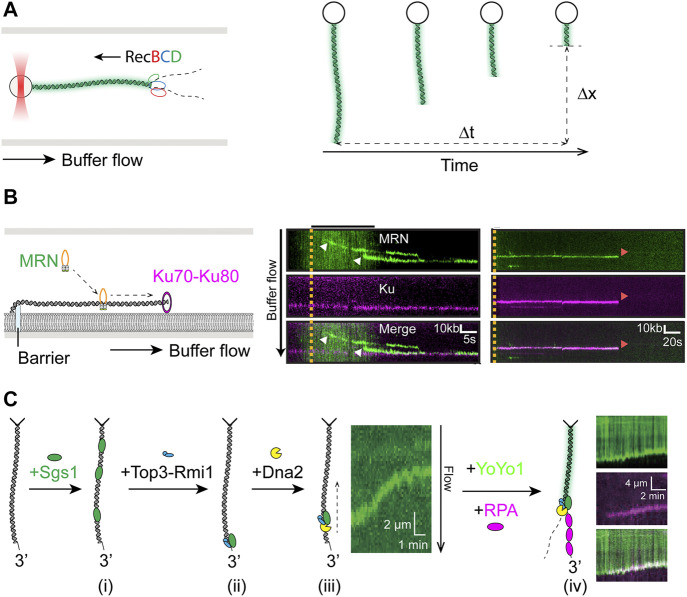
Single-molecule studies of resection in HR. **(A)** **Left:** Schematics of unlabeled RecBCD resecting YOYO1-stained DNA attached to an optically trapped bead and extended by hydrodynamic flow. **Right:** Velocity ( Δx/Δt ) and processivity of RecBCD resection could be measured by quantifying shortening of YOYO1 tract ( Δx ) over time ( Δt ). **(B)** **Left:** Schematics of fluorescently labeled MRN binding to and sliding on single-tethered dsDNA, with end-bound Ku, in a single-tethered DNA curtain assay. **Right:** Kymographs showing the Mre11-dependent nucleolytic reaction leading to MRN and Ku release from DNA. White arrows indicate MRN binding. Red arrows indicate dissociation of both MRN and Ku. Adapted from (Myler et al., 2017). **(C)** Sgs1 bound randomly to single-tethered dsDNA in DNA curtain **(i)**, but was targeted to the free ends in the presence of Top3-Rmi1 **(ii)**. Translocation (green tract in kymograph) by Sgs1-Top3-Rmi complex was only activated by addition of Dna2 **(iii)**. End resection required further addition of RPA as evident by the shortening of YOYO1-staining of dsDNA accompanied by increase of fluorescence RPA signal at the DNA end **(iv)**. Adapted from Xue et al. (2019b).

The *Escherichia coli* RecBCD is a helicase and nuclease complex that plays a critical role in repair of DSBs in bacteria by homologous recombination, whose function in promoting recombination is regulated by the Chi (χ, crossover hotspot instigator) sequence in DNA (Dillingham and Kowalczykowski, 2008; Smith, 2012). Biophysical properties of RecBCD have been extensively characterized over the past two decades using a plethora of different single-molecule techniques. Though known to be a highly processive helicase, the actions of individual RecBCD complexes had not been directly observed (Roman et al., 1992). Using YOYO-stained λ-DNA conjugated to an optically trapped bead and extended by flow (Figure 3A, left), velocity and processivity of single RecBCD enzymes were measured by quantifying the loss of YOYO signal as the dsDNA was converted to ssDNA through actions of the enzyme (Figure 3A, right) (Bianco et al., 2001). Using the same imaging technique, the mechanism of regulation for the recombination hotspot χ sequence was elegantly elucidated. Single RecBCD complexes were observed to pause precisely upon encountering the χ site and slow down afterwards, which was revealed to be due to a change in the lead helicase from RecD to RecB, rather than the loss of RecD as previously believed (Spies et al., 2003; Handa et al., 2005; Spies et al., 2007). Moving from naked DNA towards a more physiological environment, further studies on RecBCD using the high throughput DNA curtains showed that the powerful complex is capable of ejecting stably bound proteins from DNA (Finkelstein et al., 2010; Terakawa et al., 2017).

An early responder in mammalian DNA damage response, 53BP1 is an enigmatic factor that is known to prevent the formation of long 3′ overhangs by limiting 5′ end resection at DSB sites in G1 cells (Mirman and de Lange, 2020). The 53BP1-mediated block to end resection mediated is accomplished through effector proteins RIF1 and PTIP, whose recruitment depends on 53BP1 N-terminal phosphorylation by ATM (Callen et al., 2013; Zimmermann et al., 2013). For HR to proceed, restoration of end resection in S/G2 cells relies on the antagonistic functions of BRCA1 towards 53BP1 (Chapman et al., 2012; Densham et al., 2016; Hustedt and Durocher, 2016). Super-resolution light microscopy techniques such as the single-molecule localization microscopy (SMLM) has proven invaluable in elucidating the behavior of 53BP1 in response to DNA damage by ionizing radiation (Depes et al., 2018). SMLM measurements have shown cell type specific recruitment patterns of 53BP1, as well as dynamic changes in chromatin architecture, after high and low linear energy transfer irradiations (Bobkova et al., 2018; Hausmann et al., 2020).

End resection in human cells begins with the short-range resection initiated by the MRN complex with its phosphorylated cofactor CtIP, which produces a nick ∼20 nt away from the end (Anand et al., 2016; Cannavo and Cejka, 2014). Single-molecule imaging of fluorescently labeled MRN showed that the protein utilizes facilitated diffusion to reach the DNA ends (Figure 3B, left) (Myler et al., 2017). Because the NHEJ initiating factor Ku also binds tightly to DNA ends, it raises the question of how MRN behaves when encountering DNA-bound Ku, as it also relates to the problem of pathway choice in DSB repair (Scully et al., 2019). Myler et al. showed in the same study that MRN is able to release DNA-bound Ku *via* an Mre11-dependent nucleolytic reaction (Figure 3B, right), thus providing a mechanism for initiation of HR even when Ku is the first to arrive at DSB sites (Myler et al., 2017). Recent follow-up on the topic from the same groups corroborated and extended the initial finding by including CtIP and DNA-PKcs in the DNA curtain assay, showing nucleolytic release of DNA-PK by MRN/CtIP (Deshpande et al., 2020).

The short overhang generated by MRN allows long-range resection factors BLM/DNA2 or EXO1 to assemble and carry out extensive resection. Significant insights into the biophysical characteristics of these enzymes as well as their regulation have been gained from single-molecule studies. Fluorescence imaging on DNA curtains demonstrated that human and yeast Exo1 are both processive nucleases that are susceptible to displacement by multivalent ssDNA binding proteins such as RPA, though extensive resection by human EXO1 was supported by the SOSS1, another ssDNA binding complex essential for HR in human cells (Myler et al., 2016). The coordination and regulation of long-range end resection among its participants was well illustrated in a recent DNA curtain study focused on Sgs1, the yeast ortholog of BLM (Figure 3C) (Xue et al., 2019b). The authors showed that Sgs1 unwound dsDNA from internal positions in the presence of RPA (Figure 3Ci) and can be targeted to dsDNA ends in either Top3-Rmi1-dependent or independent manner (Figure 3Cii). However, Sgs1 remained inactive at DSBs until the addition of Dna2, which activated long-range translocation by Sgs1 from DNA ends (Figure 3Ciii). Furthermore, this complex lacked nucleolytic activity, which was only triggered through addition of RPA (Figure 3Civ), thus underscoring the importance of RPA in end resection as previously reported (Cejka et al., 2010a; Niu et al., 2010). Simultaneously, Sgs1 functions were also being studied using magnetic tweezers, where dsDNA unwinding initiated from a ssDNA gap with a 5′ flap produced comparable velocities to those from DNA curtain measurements, though rewinding of dsDNA, suggested to involve strand switching by Sgs1, was also observed (Kasaciunaite et al., 2019). In addition to the role in end recruitment of Sgs1 observed on DNA curtains, Top3-Rmi1 was shown to increase Sgs1 velocity when initiating translocation from flapped gap substrate, consistent with previously observed stimulatory effects (Cejka et al., 2010b; Kasaciunaite et al., 2019). The role of RPA in human RECQ helicase BLM-mediated resection was examined by two recent DNA curtain studies. BLM exhibited high speed and robustness in DNA unwinding regardless of the presence of RPA, while its end resection activity was dependent on the phosphorylation status of RPA (Xue et al., 2019a; Soniat et al., 2019). In the latter paper, resection by BLM/EXO2 or BLM/DNA2 on single-tethered DNA curtain was quantified in the presence of RPA or its phosphomimetic or phosphoblocking mutants. Phosphorylation on residues in RPA32 was found to reduce velocity and processivity of end resection by both BLM/EXO2 and BLM/DNA2, as well as inhibit their resection past individual nucleosomes, therefore acting as a negative regulator of resection (Soniat et al., 2019).

### SM Studies of Presynaptic Filament Formation and Dynamics

As the 3′-ssDNA tail is being generated by long-range resection, it is rapidly bound and protected from nucleases by single strand binding proteins, which must then be replaced by recombinases. The bacterial SSB and recombinase RecA were among the first to be studied using single-molecule methods. Efforts to characterize fundamental behavior of SSB typically favored smFRET experiments with surface-immobilized single-stranded DNA that is labeled at the ends with a donor/acceptor fluorophore pair. Wrapping of ssDNA around the SSB tetramer during binding would bring the donor and acceptor closer and allow FRET efficiency to be used as a main observable. It was elegantly shown that tetrameric SSB could spontaneously diffuse on ssDNA, capable of removing secondary structure such as a small stem-loop hairpin and promoting formation of RecA filaments (Roy et al., 2009). Coupling an optically trapped bead to the smFRET substrate to apply pN-level of force on the complex, Zhou et al. was able to discern the molecular mechanism for SSB sliding as reptation, where the motion is facilitated by the formation of a DNA bulge and its propagation around the protein opposite its direction of sliding (Zhou et al., 2011).

**FIGURE 4:**
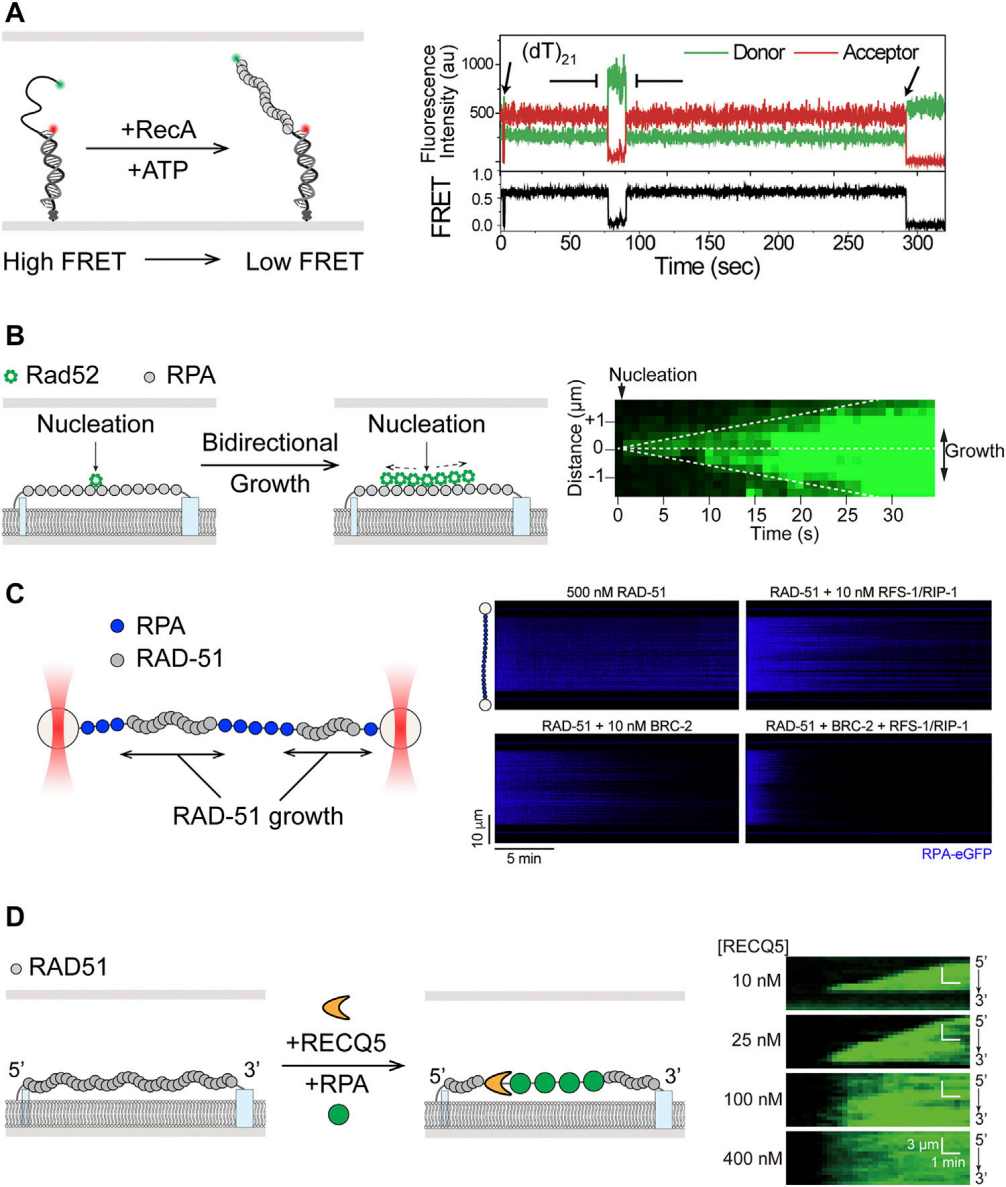
Single-molecule studies of filament assembly and dynamics in HR. **(A)** smFRET study of assembly of RecA filaments on 3′-ssDNA overhang of duplex DNA substrates, where formation of RecA filaments leads to further separation of the donor and acceptor fluorophores and lower FRET efficiency. Adapted from Joo et al. (2006). **(B)** **Left:** Single-stranded DNA curtain showing bidirectional growth of Rad52 after nucleation on unlabeled RPA-coated single stranded DNA. **Right:** Kymograph of ssDNA shows double-sided wedge shape in fluorescence signal over time. Adapted from Gibb et al. (2014b). **(C)** **Left:** Scanning confocal fluorescence imaging of ssDNA held between two optically trapped beads. **Right:** RAD-51 paralogues RFS-1/RIP-1 was shown to work synergistically with BRC-2 in stimulation of unlabeled RAD-51 filament assembly, as reflected by the loss of fluorescent RPA signal in the kymographs. Adapted from Belan et al. (2021). **(D)** **Left:** Translocation by RECQ5 on RAD51 filaments in ssDNA curtain assay, where RAD51 was displaced in the process, as shown by the increase in fluorescent RPA signal. **Right:** Kymographs showing increase of fluorescent RPA signal as RAD51 was removed by RECQ5 from ssDNA. Wedge shape growth, from 3′ to 5′, of the fluorescence signal at low RECQ5 concentrations shows the direction of translocation. Adapted from Xue et al. (2021).

Following binding of SSB to the 3′-ssDNA overhang, the *E. coli* recombinase RecA must be loaded to form a nucleoprotein filament capable of homology search and strand invasion. Observation of this process at the single-molecule level was first reported using smFRET and DNA substrates with short ssDNA overhang, where the donor/acceptor pair was placed at the junction and end of ssDNA (Figure 4A) (Joo et al., 2006). Five monomers of RecA were determined necessary for nucleation and dynamic binding and dissociation of single monomers from both ends of the filament contributed to filament growth. Notably, it was shown that RecA could displace SSB from ssDNA when a preformed nucleation cluster was present (Joo et al., 2006). Having first shown RecA binding of flow-extended double-stranded λ-DNA tethered to an optically trapped bead, Kowalczykowski et al. then reported formation of fluorescently labeled RecA filament on SSB-coated surface-tethered ssDNA (Galletto et al., 2006; Bell et al., 2012). In this work, Bell et al. observed that a RecA dimer was required for filament nucleation through titration of RecA concentration and its relationship with nucleation frequency. Using two-color labelling of RecA, it was demonstrated that RecA filament growth was bidirectional but faster in the 5′-3′ direction, consistent with previous findings (Galletto et al., 2006; Joo et al., 2006). Furthermore, *E. coli* recombination mediator proteins RecOR were shown to stimulate both RecA nucleation and filament growth (Bell et al., 2012).

Many of the same characteristics exhibited by SSB and RecA are conserved in their eukaryotic counterparts. Human RPA has been shown by smFRET to diffuse on ssDNA and melt secondary structures (Nguyen et al., 2014). Dynamics of RPA filament were thoroughly investigated using single-stranded DNA curtains (Gibb et al., 2012; Ma et al., 2017b). RPA filament formed on ssDNA was shown to be stable for over 2 h when unbound proteins were flushed out (Gibb et al., 2014a; Deng et al., 2014; Ma et al., 2016). When challenged with free protein in solution, it was observed that ssDNA-bound RPA could be exchanged with those in solution in a manner dependent on concentrations of the free RPA, consistent with facilitated dissociation previously reported for DNA binding proteins with multiple contacts (Graham et al., 2011; Gibb et al., 2014a; Deng et al., 2014; Ma et al., 2016). Direct visualization of fluorescently labeled human RAD51 showed conserved end-biased bidirectional filament growth on dsDNA, initiated by nucleation of ∼2–3 monomers (Hilario et al., 2009). Assembly of RAD51 filament on the physiologically relevant RPA-coated DNA as well as its disassembly characteristics were also recapitulated on ssDNA curtains (Ma et al., 2016).

To overcome RPA-mediated inhibition of RAD51 filament formation, mediator proteins such as BRCA2 and RAD51 paralogs are needed (Bonilla et al., 2020). Effects of yeast Rad52, considered a possible functional ortholog of human BRCA2, on the dynamics of presynaptic filaments were revealed using ssDNA curtains (Figure 4B, left) (Gibb et al., 2014b). Fluorescently labeled Rad52 was shown to nucleate on RPA-coated ssDNA and promote bidirectional growth for additional Rad52 binding (Figure 4B, right). Rad52-RPA clusters were also observed to remain after formation of extended Rad51 filaments and served as nucleation sites for additional binding of RPA and Rad52 (Gibb et al., 2014b). Recent smFRET work suggests that the Rad52 destabilizes the DBD-D DNA binding domain of RPA, thereby increasing access to ssDNA previously occluded by RPA (Pokhrel et al., 2019). Many of these same characteristics of Rad52 were also recapitulated in a DNA curtain study of human RAD52, whose deletion in vertebrates does not produce a strong phenotype, with the exception that human RAD52 and RPA could not rebind to remaining clusters after assembly of human RAD51 filaments (Ma et al., 2017a). In addition, effects of RAD51 paralogs on presynaptic filaments have been the subject of several recent single-molecule studies. In a smFRET study of *C. elegans* RAD-51 paralogs RFS-1/RIP-1, surfaced-immobilized substrates were labeled with donor and acceptor dyes seven nucleotides apart in the ssDNA region (Taylor et al., 2015). Addition of RAD-51 to naked ssDNA led to transition from high FRET to low FRET, reflecting the stretching of ssDNA upon RAD-51 binding. RFS-1/RIP-1 bound RAD-51 filament exhibited intermediate FRET value along with broadening of the FRET signal distribution, suggesting that these paralogs remodeled RAD-51 filaments to a more flexible conformation (Taylor et al., 2015). Most recently, optical tweezers with confocal fluorescence imaging (Lumicks C-trap) and DNA curtains were separately applied to better understand the actions of RFS-1/RIP-1 and the yeast Rad51 paralogs Rad55-Rad77, respectively (Belan et al., 2021; Roy et al., 2021). Both studies showed that the paralogs promote RAD51 filament assembly through transient interactions, dissociating rapidly by hydrolyzing ATP. In addition, Belan et al. found that RFS-1/RIP-1 synergize with BRC-2 (human BRCA2 homolog) in promoting presynaptic filament assembly, specifically by engaging with the 5′ end of the RAD-51 filament to stimulate growth in a 3′→5′ direction (Figure 4C) (Belan et al., 2021). Roy et al. also showed that Rad55-Rad57 antagonism of anti-recombinase Srs2 might be through promoting faster re-assembly of Rad51 rather than inhibiting the anti-recombinase itself, as previously suggested (Liu J. et al., 2011; Roy et al., 2021).

Excessive recombination, also referred to as hyper-recombination, however, can be genotoxic and must be prevented. Counteracting the effects of RAD51 mediators that promote filament assembly are the negative regulators, or anti-recombinases. Several SF1 helicases are known to display anti-recombinase activity. Bacterial UvrD and PcrA have been implicated in dismantling of RecA filaments in genetic and biochemical experiments (Veaute et al., 2005; Bidnenko et al., 2006; Lestini and Michel, 2007; Petrova et al., 2015). In particular, smFRET studies showed that PcrA strips RecA filaments off DNA through a reeling motion (Park et al., 2010). The yeast SF1 helicase Srs2 has also been shown to prevent recombination by dismantling the Rad51-ssDNA nucleofilament through stimulation of ATP hydrolysis by Rad51 and its dissociation (Krejci et al., 2003; Veaute et al., 2003; Antony et al., 2009). Actions of Srs2 on different HR intermediates have been visualized at the single-molecule level. In one smFRET study, Srs2 cleared Rad51 bound to short ssDNA overhangs and exhibited repetitive motion at the ssDNA/dsDNA junction, proposed to prevent reformation of Rad51 filament (Qiu et al., 2013). Single-stranded DNA curtain experiments showed that Srs2 was capable of processively translocating on naked ssDNA, as well as RPA-coated ssDNA, Rad51-ssDNA, and ssDNA bound by both RPA and Rad52 (De Tullio et al., 2017; Kaniecki et al., 2017). While translocating on protein-bound ssDNA, Srs2 also efficiently removed RPA, Rad51, Rad52, and short heteroduplexes formed with Rad51. Remarkably, this robust anti-recombination function of Srs2 was strongly inhibited by the presence of meiosis-specific recombinase Dmc1 within the presynaptic filament (Crickard et al., 2018).

In addition to SF1 helicases, members of the RecQ subfamily of SF2 helicases have also been implicated in anti-recombination functions (Branzei and Szakal, 2017; Larsen and Hickson, 2013). A DNA curtain study showed that Sgs1, apart from its role in end resection, also acted on presynaptic filaments (Crickard et al., 2019). Sgs1 was observed translocating on RPA-coated ssDNA and, in accordance with its expected anti-recombinase activity, displacing Rad51 while translocating on the Rad51-ssDNA filament. Sgs1-mediated Rad51 removal was found to be independent of Rad51 ATP hydrolysis, in stark contrast to the mechanism employed by Srs2 (Antony et al., 2009; Kaniecki et al., 2017). Though similar to the case of Srs2, Sgs1 action was also inhibited by Dmc1 (Crickard et al., 2019). Functional conservation of RECQ helicases in anti-recombination was recently demonstrated for the human RECQ5 on DNA curtain (Figure 4D, left) (Xue et al., 2021). RECQ5 not only translocated on ssDNA bound by RPA, RAD51, or DMC1, but also removed these proteins in the process (Figure 4D, right). Real-time observation of RAD51 removal by RECQ5 is consistent with previous results from biochemical assays (Hu et al., 2007). Similar to Sgs1, RECQ5 was able to strip ATPase-deficient RAD51 from ssDNA, suggesting a mechanism not coupled to RAD51 ATP hydrolysis. The ability of RECQ5 to translocate and disrupt DMC1 filaments contrasts with the inhibitory effects of Dmc1 on Sgs1 and Srs2, suggesting that it may play a role in meiosis (Xue et al., 2021). Finally, as mentioned above in the context of DNA end resection, BLM showed robust dsDNA unwinding but little interaction with RPA- or active ATP-bound RAD51-coated ssDNA in DNA curtain assays, even though it was considered an anti-recombinase capable disrupting inactive ADP-bound RAD51 filaments (Bugreev et al., 2007; Xue et al., 2019a). The apparent differences in the abilities of RECQ5 and BLM to interact with different HR intermediates may arise from differences in protein domain architecture and reflect division of labor among RECQ helicases in HR.

### SM Studies of Homology Search

**FIGURE 5:**
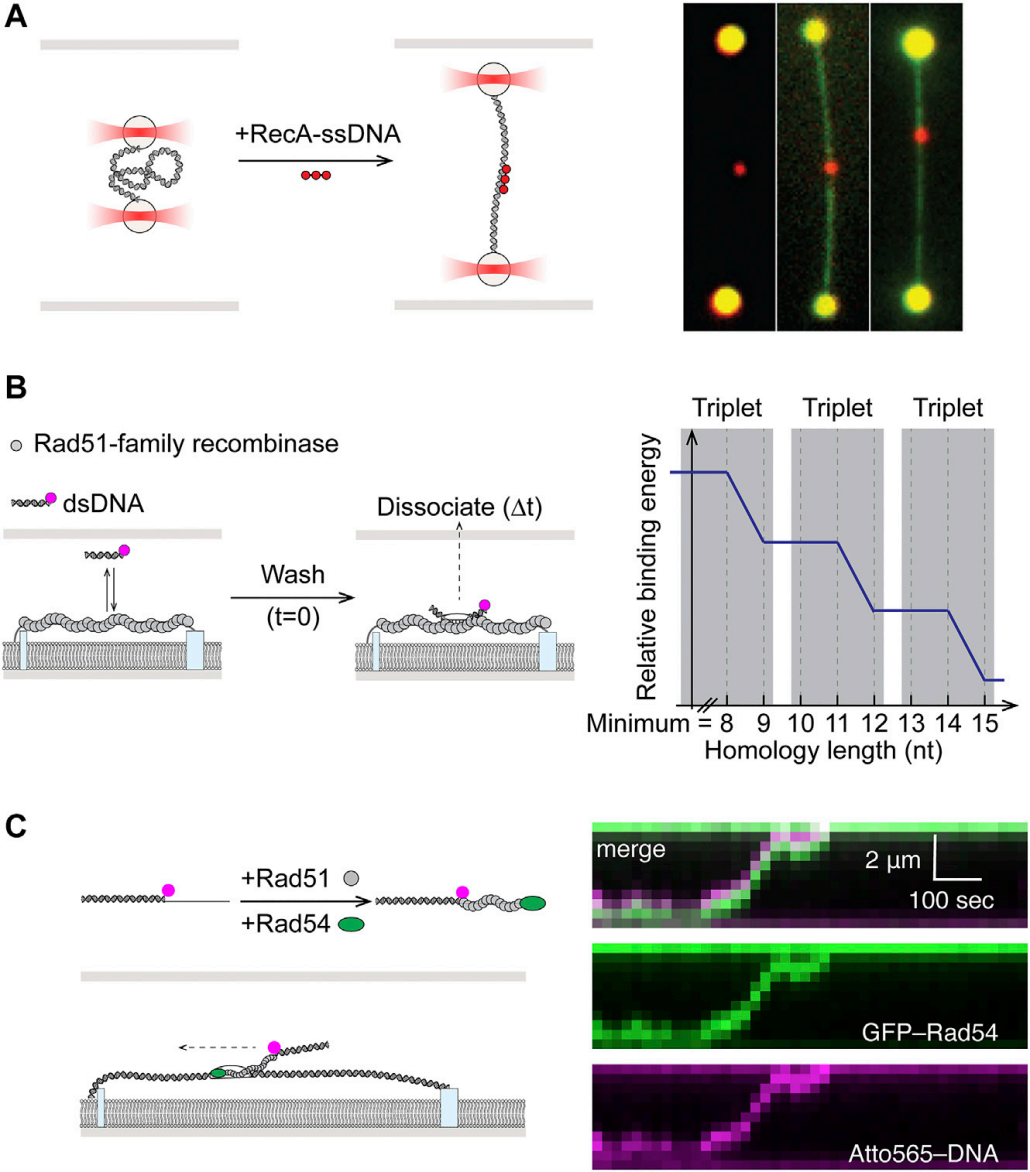
Single-molecule studies of homology search in HR. **(A)** **Left:** Dual optical trap setup allowed precise control of dsDNA end-to-end distance in study of homology search by RecA. **Right:** Fluorescent RecA-ssDNA bound to expected homology positions in dsDNA after incubation. dsDNA could be visualized by staining with YOYO1. Adapted from Forget and Kowalczykowski (2012). **(B)** Fluorescently-labeled and microhomology-containing dsDNA fragments were incubated with nucleofilaments formed by Rad51-family recombinases on ssDNA curtain. Dissociation times of bound-particles post-wash ( Δt ) showed energy stabilization occurring in steps of Watson-Crick base triplets. **(C)** **Left:** Presynaptic complexes were assembled by mixing Rad51 and Rad54 with dye-labeled partial duplex DNA containing homology in the ssDNA overhang region. **Right:** Dual color imaging of labeled DNA and Rad54 showed that Rad54 drove active homology search along DNA in an ATP hydrolysis-dependent manner. Adapted from Crickard et al. (2020).

Once the stable presynaptic filament forms, it must locate sequence homology elsewhere in the genome, against a vast background of heterologous sequence. The recombinase then catalyzes a strand exchange reaction to form a heteroduplex containing the ssDNA base paired with the complementary strand and displacing the strand containing the homologous sequence (D-loop). At its core, the homology search process is highly similar to target search by other ubiquitous sequence specific DNA-binding proteins. A theoretical solution to the target search problem has been known for four decades as facilitated diffusion (Berg et al., 1981). Understanding of this process, involving a combination of 1D and 3D diffusion as well as microscopic hopping and intersegmental transfer, has seen significant contributions from single-molecule experiments. Following their earlier work on RecA filament assembly, the Kowalczykowski and Ha groups were the first to shed light on the mechanism behind homology search by RecA presynaptic filaments (Forget and Kowalczykowski, 2012; Ragunathan et al., 2012). In the first study, dsDNA serving as the homologous sequence donor was held between two optically trapped beads in a “dumbbell” configuration (Figure 5A). By systematically varying the distance between the beads, hence the contour length and 3D conformation of the DNA, it was shown that RecA filaments conducted homology search via multiple weak contacts for sampling DNA sequence within a 3D volume, a mechanism the authors termed “intersegmental contact sampling” (Figure 5A) (Forget and Kowalczykowski, 2012). In the second smFRET study, free dsDNA homology donor and surface-immobilized RecA-ssDNA were labeled with donor and acceptor dyes, respectively. By observing the dynamic FRET values while controlling for sequence homology, a sliding model was proposed, in which RecA filaments can diffuse along the dsDNA track while efficiently sampling for homology as short as six nucleotides (Ragunathan et al., 2012). Together these studies demonstrate that homology search by RecA filaments occurs through facilitated diffusion using a combination of 1D sliding and 3D diffusion, expedited *via* intersegmental contact sampling.

More detailed understanding of minimum sequence homology requirements and kinetics of sampling were uncovered in a pair of papers using single-stranded DNA curtains (Lee et al., 2015; Qi et al., 2015). In these experiments, presynaptic filaments were assembled on long ssDNA tethered to the lipid bilayer surface, while fluorescently labeled duplexes containing varying degrees of homology were free in solution (Figure 5B). The first study revealed that eight nucleotides of homology was the minimum requirement for recognition by Rad51 and stable capture, while subsequent strand exchange occurred in precise three nucleotide steps (Qi et al., 2015). The follow-up work illustrated that the base triplet stepping for homology recognition (Figure 5B) was a conserved feature in the RecA family of recombinases from RecA to Rad51, including the meiosis-specific Dmc1. Dmc1, however, was also unique in its ability to stabilize internal mismatches. Whereas mismatches in RecA or Rad51 filaments could be tolerated but would not contribute to stabilizing recognition complex (Lee et al., 2015).

Adding to the complexity of homology search is the fact that the process in eukaryotic cells also involves the multi-functional SWI2/SNF2 motor proteins Rad54 and Rdh54 (Ceballos and Heyer, 2011). Early single-molecule work had shown that both Rad54 and Rdh54 are highly processive translocases on dsDNA (Amitani et al., 2006; Nimonkar et al., 2007; Prasad et al., 2007). While Rad54 is also known to facilitate Rad51-mediated homologous DNA pairing *in vitro* and homology search *in vivo*, its exact mechanism of action remained unknown (Petukhova et al., 1998; Renkawitz et al., 2013). Recently, using double-tethered dsDNA curtains as sequence donor and labeled partial duplex DNA with 3′ Rad51-ssDNA filament, Rad54 was shown to promote targeting to homologous DNA by translocating with the presynaptic filament on dsDNA (Figure 5C) (Crickard et al., 2020). This ATP-dependent behavior adds to the 3D diffusion mechanism of the homology search and serves in reducing dimensionality and increasing search efficiency. Moreover, while driving active translocation, Rad54 induced transient strand opening coupled to RPA binding, potentially allowing the Rad51 presynaptic complex to sample both strands of dsDNA donor for homology (Crickard et al., 2020).

## Overview of NHEJ

Upon formation of a DSB, the “canonical” NHEJ pathway proceeds through three distinct steps: synapsis, end processing, and ligation (Figure 2B). The DNA ends are first recognized by Ku70-Ku80, a ring-shaped heterodimer with high affinity to DNA ends (Walker et al., 2001). After binding, Ku70-Ku80 (Ku) serves as a ‘tool belt’ that interacts and stabilizes many subsequence NHEJ proteins (Lieber, 2008). One of the first factors recruited to DNA-bound Ku is DNA-PKcs (DNA-dependent protein kinase, catalytic subunit), a member of the phophoinositide 3-kinase family (Gottlieb and Jackson, 1993; Smith and Jackson, 1999; Falck et al., 2005). Together they form the DNA-PK holoenzyme, whose kinase activity is required for NHEJ, as it phosphorylates many other NHEJ accessory factors as well as itself (Uematsu et al., 2007; Jette and Lees-Miller, 2015; Jiang et al., 2015). In the next step, the two broken DNA ends must be brought to close proximity to enable synapsis in a dynamic process. The mechanism of synapsis depends on binding of LIG4 (DNA ligase IV), XRCC4, and XLF (XRCC4-like factor) (Stinson and Loparo, 2021). LIG4 and XRCC4 form an active complex, through interactions between XRCC4 and the region between the BRCT motifs in the C-terminal of LIG4 (Critchlow et al., 1997; Grawunder et al., 1997; Grawunder et al., 1998; Sibanda et al., 2001; Wu et al., 2009). XLF was identified to interact with LIG4-XRCC4 to promote NHEJ (Ahnesorg et al., 2006; Buck et al., 2006). Evidence also suggests that XRCC4 and XLF may form filaments that help bridge DNA ends (Hammel et al., 2011; Ropars et al., 2011; Andres et al., 2012; Mahaney et al., 2013). Post synapsis, blunt ends that do not require further processing may be ligated directly by XRCC4-LIG4. However, naturally occurring DSBs typically have incompatible ends that cannot be readily ligated. Therefore, end processing in the forms of resection by nucleases and/or addition and filling-in by the X family of DNA polymerases are often needed before generating compatible ends for ligation (Waters et al., 2014; Chang et al., 2016). Artemis is a nuclease associated with NHEJ and essential for V(D)J recombination (Ma et al., 2002; Riballo et al., 2004). Its C-terminal region has been found to interact with LIG4 and DNA-PKcs (Niewolik et al., 2006; De Ioannes et al., 2012; Malu et al., 2012). While members of the X family DNA polymerase, pol λ, pol μ, and TdT (terminal deoxynucleotidyl transferase) are all implicated in NHEJ with different levels of template dependence (Nick McElhinny et al., 2005). Recruitment of these polymerases to sites of NHEJ is known to occur through interactions with Ku and XRCC4-LIG4 via their N-terminal BRCT domain (Mahajan et al., 2002; Fan and Wu, 2004; Ma et al., 2004). Notably, recent structural evidence has indicated that synapsis of DNA ends with single nucleotide homology could be mediated solely by TdT or pol μ, in the absence of other NHEJ core factors (Kaminski et al., 2020; Loc’h et al., 2016). In cases of unligatable chemical blocks at DNA ends, PNKP (polynucleotide kinase 3′-phosphatase), aprataxin and PNKP-like factor (APLF), or tyrosyl-DNA phosphodiesterase 1/2 (TDP1/2) may be recruited to DSB sites for processing (Zhao et al., 2020a). Finally, PAXX (paralogue of XRCC4 and XLF) is a recently discovered factor that promotes ligation and assembly of core NHEJ proteins (Ochi et al., 2015; Xing et al., 2015). Although its functions in NHEJ appear to overlap with those of XLF (Tadi et al., 2016).

## Single-Molecule Studies of NHEJ

### Fundamental Mechanism of Synapsis

Synapsis is the step in which the two broken DNA ends are brought together to close proximity such that NHEJ machinery may assemble in a stable complex and assess the actions needed to restore the structural integrity of DNA. Detailed mechanistic insights on this critical early step are therefore prerequisite to understanding of the pathway. The dynamic nature of the process involving two DNA ends has made smFRET the single-molecule platform of choice in studying the system. By measuring the fluorescence energy transfer between the donor-labeled surface-immobilized fragment and the acceptor-labeled freely-diffusing fragment, smFRET allows real-time monitoring of intermolecular synapsis. High FRET indicates close proximity of the two DNA ends, while fluctuating FRET values would suggest dynamics in the process of DNA end alignment.

**FIGURE 6:**
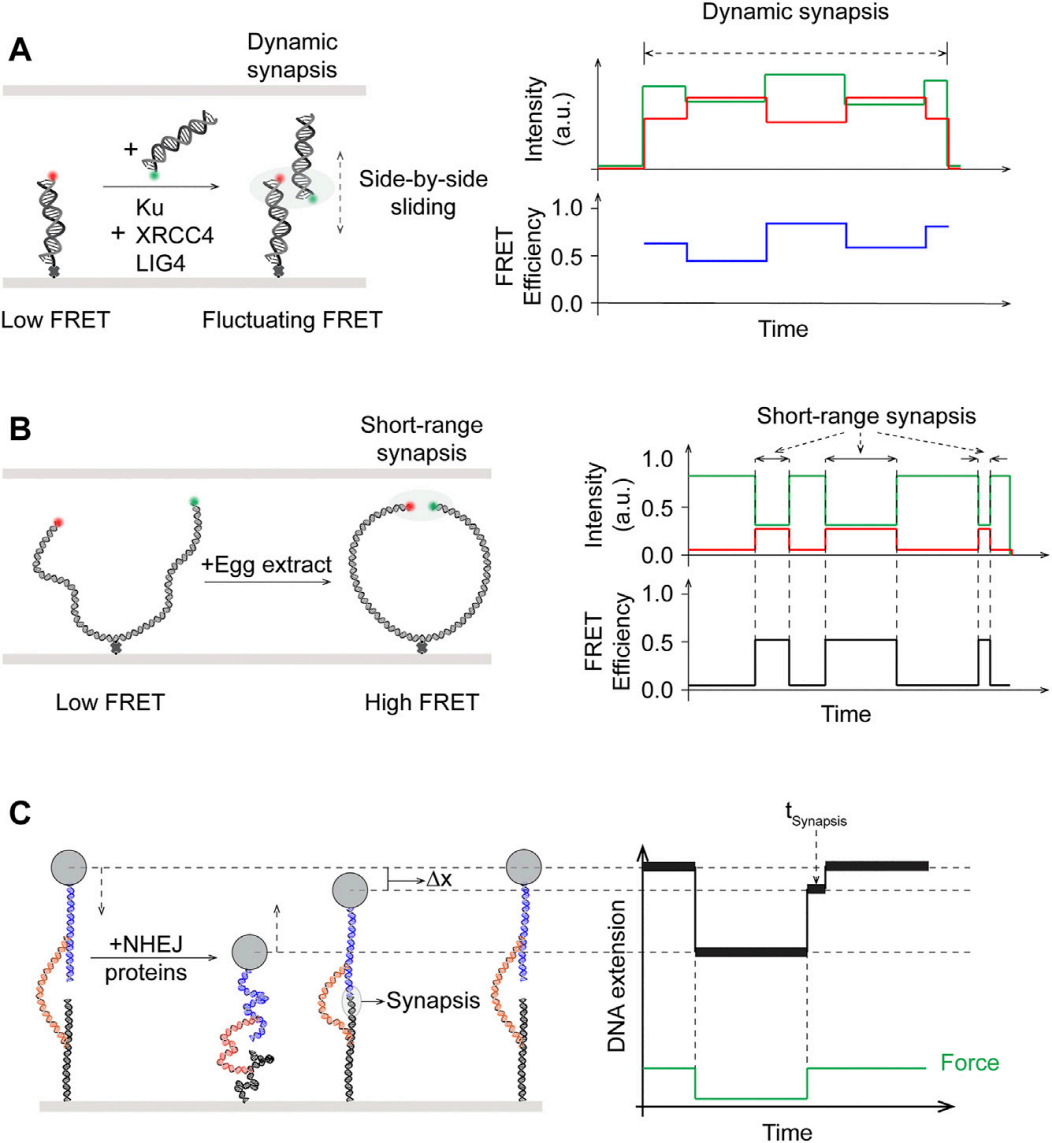
Single-molecule studies of end synapsis in NHEJ. **(A)** **Left:** Schematics of intermolecular smFRET showing synapsis upon addition of purified human Ku, XRCC4, LIG4, but in the absence of DNA-PKcs, to be dynamic with fluctuating and widely distributed FRET efficiency, suggesting that the two broken ends may slide relative to and past each other during synapsis. **Right:** Side-by-side sliding of the donor with respect to the acceptor could give rise to the fluctuating FRET efficiency values. **(B)** **Left:** Schematics of intramolecular smFRET used to monitor end synapsis mediated by NHEJ factors in egg extract. **Right:** Distinct high FRET state reverted back to low FRET state, showing short-range synapsis was dynamic. **(C)** Magnetic tweezers with novel DNA substrate design tracks DNA extension and enables measuring dwell times ( Δt ) of transient synapsis, by cycling between low force/extension that allows formation of synaptic complex and high force/extension to disrupt synapsed but unligated ends. A change in DNA extension ( Δx ) at the same high force is observed when the synapsed ends are disrupted.

Early models derived from work using purified proteins in bulk biochemical assays, electron microscopy, and x-ray scattering as well as laser microirradiation of cells followed by immunofluorescence imaging suggested that DNA-PKcs is recruited by Ku to DNA breaks and together they are able to bridge the broken DNA ends (DeFazio et al., 2002; Hammel et al., 2010b; Kim et al., 2005; Weterings et al., 2003). Nonetheless, the lack of spatiotemporal resolution precluded these studies from revealing any transient intermediate steps or subcomplexes in the process. Using smFRET with two DNA fragments containing four nucleotide homology at the ends and differentially labeled with donor and acceptor fluorophores, Rothenberg and coworkers observed co-localization of the donor/acceptor pairs after addition of purified NHEJ components except DNA-PKcs (Figure 6A, left) (Reid et al., 2015). Although aggregated joining was observed in the presence of DNA-PKcs, this result cast doubt over the requirement of DNA-PKcs in synapsis. This end joining process mediated by Ku70-Ku80, XRCC4-LIG4, and XLF was revealed to be dynamic, as shown by FRET efficiency distributions (Figure 6A, right). These distributions exhibited widths indicative of the possibility that the DNA ends may be positioned in a side-by-side manner, in addition to end-to-end. Examination of fluctuating FRET trajectories in conjunction with using substrates that varied in end chemistry also supported the notion of DNA ends in “adjacent configuration” during the synaptic process that is highly dynamic (Figure 6A). More mechanistic details were uncovered in a later follow-up smFRET study by the Rothenberg and Lieber laboratories (Zhao et al., 2019). It was shown that the first “flexible” stage of blunt end synapsis (FS), mediated by Ku and XRCC4-LIG4, involves the dsDNA ends being brought into a parallel side-by-side configuration where they can still slide along each other, as evidenced by fluctuating FRET efficiency values. Flexible synapsis, shown to be independent of DNA-PKcs, can then be converted to a close synaptic state (CS) by XLF or PAXX, where the two DNA ends are aligned in close proximity in an end-to-end manner.

The lack of DNA-PKcs requirement in synapsis as monitored by smFRET contradicts existing evidence for its role in NHEJ *in vivo* (Baumann and West, 1998; Cottarel et al., 2013; Jiang et al., 2015; Zhao et al., 2006). This apparent discrepancy was further investigated by Loparo and coworkers using smFRET and cell-free extract of *X. laevis* eggs (Figure 6B) (Graham et al., 2016). Xenopus egg extract represents a more physiological system compared to biochemical reconstitution of purified proteins, and has been established for single-molecule imaging studies as well as being capable of Ku- and DNA-PK-dependent DNA end joining (Di Virgilio and Gautier, 2005; Labhart, 1999; Yardimci et al., 2012). In addition to intermolecular synapsis of two separate DNA fragments, a longer 2 kbp DNA fragment with donor/acceptor-labeled blunt ends and an internal biotin for surface immobilization was used in this study to facilitate intramolecular end joining (Figure 6B, left) (Graham et al., 2017). Based on the distance between the donor/acceptor dyes, synapsis was observed to occur through two distinct stages: long-range (LR) where both dyes were present but no FRET, and short-range (SR) where FRET was seen between the dye pair (Figure 6B, right). In contrast to previous single-molecule work, LR synapsis in Xenopus egg extracts required both Ku70-Ku80 and DNA-PKcs, though the kinase activity of the latter is not needed. Transition from LR to SR synapsis would occur after several seconds and require the catalytic activity of DNA-PK, as well as the presence of XRCC4-LIG4 and XLF, though not the catalytic activity of LIG4.

Unlike order of assembly studies, quantifying biophysical observables such as step-wise reaction energetics has mostly been intractable for bulk biochemistry. In particular, a novel DNA substrate featuring two free DNA ends tethered *via* a leash held by magnetic tweezers has been developed as a unique single-molecule force spectroscopy approach to probe the energetics of NHEJ synapsis with reconstitution of purified proteins (Kostrz et al., 2019; Wang et al., 2018). By cycling between low and high forces on a single tether and monitoring changes in tether length, Strick and coworkers demonstrated that Ku and DNA-PKcs are required to first establish a brief (∼100 ms) stage of synapsis of DNA ends (Wang et al., 2018) (Figure 6C). This initial step is further stabilized by either XRCC4-LIG4 and XLS and/or PAXX, each contributing *k*_*B*_ *T*-scale energy, leading to long-lived (∼seconds) intermediate stages and stable (∼minute) synaptic complexes. Notably, these results support the two distinct stages of synapsis observed by Graham et al. using smFRET. The subcomplex containing Ku and DNA-PKcs and stabilized by PAXX (∼2 s) appears consistent with the long range synapsis, while the full complex further stabilized by XRCC4-LIG4 and XLF (∼66 s) would correspond to the short range synapsis (Stinson and Loparo, 2021). Most recently, the same technique was applied to demonstrate the dynamic properties of prokayrotic NHEJ synapsis involving just the Ku heterodimer and Ligase D (Oz et al., 2021). Although debates remain regarding whether DNA-PKcs is required for synapsis, as the results appear to be dependent on the system employed, recent single-molecule work have unambiguously shown the process to be a dynamic process with distinct stages.

### Roles of XLF in Synaptic Complexes

**FIGURE 7:**
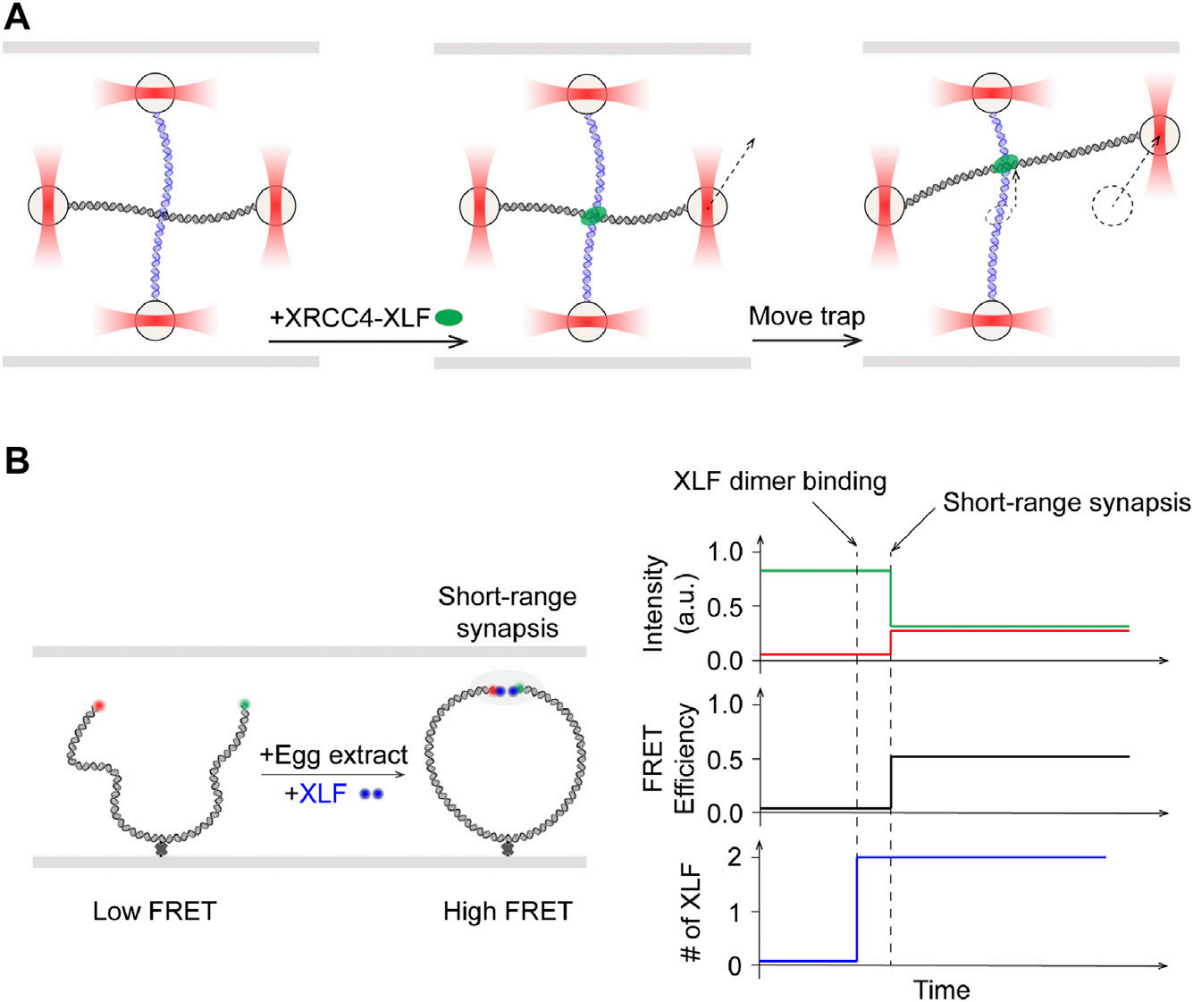
Single-molecule studies of roles of XLF in NHEJ synapsis. **(A)** **Left:** Quad optical trap was used to show bridging of two separate DNA molecules by XRCC4-XLF. **Middle:** One trap was moved to shift one of the two DNA molecules. **Right:** XRCC4-XLF was able to slide while maintaining the bridge. **(B)** **Left:** Schematics of smFRET study using fluorescently labeled XLF dimer with egg extract. **Right:** Onset of high FRET, indicative of short-range synapsis between the DNA ends, was preceded by increase in fluorescence signal corresponding to binding of one XLF dimer.

XRCC4-like factor (XLF, or Cernunnos) is identified as an interactor of XRCC4 and regulator of ligation (Ahnesorg et al., 2006; Buck et al., 2006). X-ray crystallography and electron microscopy studies have shown that XLF and XRCC4 can form filaments in crystals (Andres et al., 2012; Hammel et al., 2010a; Mahaney et al., 2013; Ropars et al., 2011). Filamentous structures of XRCC4, XLF, and LIG4 have also been observed at DSB sites using super-resolution fluorescence microscopy (Reid et al., 2015). The mode of interaction between XLF-XRCC4 complexes and DNA remained elusive until the collaborative work from the Modesti, Peterman, and Wuite groups. In a single-molecule tour de force, dual- and quadruple optical traps were combined with wide-field fluorescence imaging to demonstrate that XRCC4-XLF complexes robustly bridged two independent DNA fragments (Brouwer et al., 2016). These complexes acted like sleeves that were able to withstand high applied forces and capable of sliding along DNA molecules (Figure 7A). Specific contributions from XLF in synapsis in the presence of other NHEJ core proteins were elucidated using smFRET (Figure 7B, left). Mutagenesis in XLF and XRCC4 showed that close alignment of donor/acceptor dye labeled DNA ends in the xenopus egg extracts system required interactions between these two proteins (Graham et al., 2018). Moreover, binding of a single dye-labeled XLF dimer was sufficient to mediate this short-range synapsis, which is shown to also be dependent on interactions of both XLF head domains with XRCC4 (Figure 7B, right). These findings call into question the requirement and relevance of XLF-XRCC4 filaments, as observed in bulk, in NHEJ. More corroborating evidence incompatible with the XLF filament hypothesis emerged in a subsequent smFRET study by Rothenberg and Lieber laboratories using reconstituted human NHEJ proteins. XLF was found to drive DNA ends into close proximity in a manner that is not strongly dependent on XLF concentrations, suggesting that only one to a few XLF dimers are needed at the DNA ends (Zhao et al., 2019).

### End Processing and Ligation

Many DNA ends at DSB sites, regardless of their origins, require end processing before repair. The arsenal of NHEJ end-processing enzymes include nucleases, polymerases, kinases, phosphatases, and phosphodiesterases (Chang et al., 2017). The effects of chemically diverse DNA ends have on the dynamics of how they come together during synapsis is a question uniquely suited for single-molecule studies. Pairing efficiency as monitored by smFRET was shown to be strongly affected by phosphorylation status of the 5′ end of compatible DNA ends with four nucleotide overhangs in a minimal reconstituted system (Reid et al., 2017). Two distinct kinetic regimes, transient (<5 s) and persistent (>30 s), were found to exist for end pairing during the process, and that their energetics are modulated by the 5′ phosphate, through recognition by LIG4. In the absence of other end processing factors in this single-molecule work, a model involving an iterative process was proposed, where incompatible ends within a synaptic complex would fall apart to provide access by the processing enzymes and thus generating new compatible ends for synapsis and ligation (Reid et al., 2017). Subsequent smFRET work further examined the ability of LIG4 to sense complex ends in the minimal reconstituted system. At DNA ends with overhangs containing varying degrees of complementarity, LIG4 was shown to promote alignment of complementary ends in pre-catalytic positions, but allow dynamic sampling of alignments for terminal mismatches or ends with embedded complementarity that requires nucleolytic end process before ligation (Conlin et al., 2017). While the above-mentioned work demonstrated the participation of LIG4 in the alignment of DNA ends, in order to understand how end processing is coordinated with alignment of DNA, simultaneous observation of end processing enzymes and synapsis would be required. Taking advantage of the xenopus egg extracts system, Stinson et al. recently expanded on the requirement of LIG4-mediated close alignment of the DNA ends and showed that end processing is coordinated to take place within this synaptic complex (Stinson et al., 2020). Synaptic complex formation was monitored through smFRET with donor/acceptor dye-labeled DNA ends as before. To observe pol λ activity, the first incoming nucleotide was labeled with a fluorescent quencher, which once incorporated leads to quenching of the donor fluorophore. To observe Tdp1 activity, one of the 3′ adducts is conjugated to the donor fluorophore, which once processed by Tdp1 will be lost. It was observed that donor signal loss was preceded by high FRET, indicating close alignment of DNA end (Stinson et al., 2020). These data clearly demonstrated that end processing by pol λ and Tdp1 occurs within the short-range synaptic complex. This level of coordination between end processing and ligation during synapsis has thus been proposed as a regulatory mechanism to minimize errors and maximize fidelity of NHEJ (Stinson et al., 2020; Stinson and Loparo, 2021). Finally, attesting to the flexibility of NHEJ, it was recently reported in a smFRET study that pol μ, another X family polymerase participating in NHEJ alongside pol λ, alone can mediate synapsis of 3′ overhangs with at least 1 nt homology, in the absence of Ku (Zhao et al., 2020b).

## Conclusion and Perspectives

Single-molecule techniques have advanced and matured by leaps and bounds, thanks to technological improvements in equipment and reagents such as cameras and fluorescent dyes. The field has also expanded and benefited from commercialization of single-molecule instruments. An underlying technical challenge in single-molecule work has always been to achieve higher spatial and temporal resolutions. And this drive has steadfastly pushed technical innovations in the field. As single-molecule studies are typically built with a bottom-up approach, the field is constantly striving for increased levels of complexity in biological systems under examination. For mechanistic studies of homologous recombination, challenges remain, including but not limited to, in addressing functions of RAD51 mediator proteins and incorporating other accessory proteins in reconstituting the process from filament assembly to strand invasion, among others. Studying repair in general within the physiologically relevant context of chromatin has also proven challenging. Since multiple repair pathways exist and are available to cells for DSB repair, pathway choice is an overarching subject that bridges studies of individual repair mechanisms. Though initial work exists, the molecular mechanism for how competing repair mechanisms cooperate at the single-molecule level has largely been elusive. Biochemical reconstitutions of repair using purified recombinant proteins provide a clear, pre-defined set of parameters and have been the preferred system for single-molecule studies. However, functional cell extracts that already contain the proteins of interest may be the key to the pursuit of higher degrees of reaction complexity.

The unprecedented level of detail in mechanistic insights from single-molecule experiments may at times be seemingly at odds with existing biochemical or *in vivo* evidence and require careful reconciliation. It is worth repeating that gaps exist among these approaches, such that a comprehensive picture is best constructed when all evidence is considered together. Indeed, differences exist even between comparable single-molecule studies using the same techniques, resulting in apparently incompatible interpretation of the mechanism. Building on existing imaging platforms that focus on studies of particular stages of the process, further developments of single-molecule imaging *in vivo*, complemented by biochemical and *in vitro* studies, will undoubtedly help uncover deeper understanding of DSB repair.
